# Understanding the role of the kynurenine pathway in human breast cancer immunobiology

**DOI:** 10.18632/oncotarget.6467

**Published:** 2015-12-04

**Authors:** Benjamin Heng, Chai K. Lim, David B. Lovejoy, Alban Bessede, Laurence Gluch, Gilles J. Guillemin

**Affiliations:** ^1^ Department of Biomedical Sciences, Faculty of Medicine and Health Sciences, Macquarie University, Sydney, NSW, Australia; ^2^ ImmuSmol, Pessac, France; ^3^ The Strathfield Breast Centre, Strathfield, NSW, Australia

**Keywords:** breast cancer, kynurenine pathway, immune-evasion

## Abstract

Breast cancer (BrCa) is the leading cause of cancer related death in women. While current diagnostic modalities provide opportunities for early medical intervention, significant proportions of breast tumours escape treatment and metastasize. Gaining increasing recognition as a factor in tumour metastasis is the local immuno-surveillance environment. Following identification of the role played by the enzyme indoleamine dioxygenase 1 (IDO1) in mediating maternal foetal tolerance, the kynurenine pathway (KP) of tryptophan metabolism has emerged as a key metabolic pathway contributing to immune escape. In inflammatory conditions activation of the KP leads to the production of several immune-modulating metabolites including kynurenine, kynurenic acid, 3-hydroxykynurenine, anthranilic acid, 3-hydroxyanthranilic acid, picolinic acid and quinolinic acid. KP over-activation was first described in BrCa patients in the early 1960s. More evidence has since emerged to suggest that the IDO1 is elevated in advanced BrCa patients and is associated with poor prognosis. Further, IDO1 positive breast tumours have a positive correlation with the density of immune suppressive Foxp3+ T regulatory cells and lymph node metastasis. The analysis of clinical microarray data in invasive BrCa compared to normal tissue showed, using two microarray databank (cBioportal and TCGA), that 86.3% and 91.4% BrCa patients have altered KP enzyme expression respectively. Collectively, these data highlight the key roles played by KP activation in BrCa, particularly in basal BrCa subtypes where expression of most KP enzymes was altered. Accordingly, the use of KP enzyme inhibitors in addition to standard chemotherapy regimens may present a viable therapeutic approach.

## BREAST CANCER

**Figure 1:**
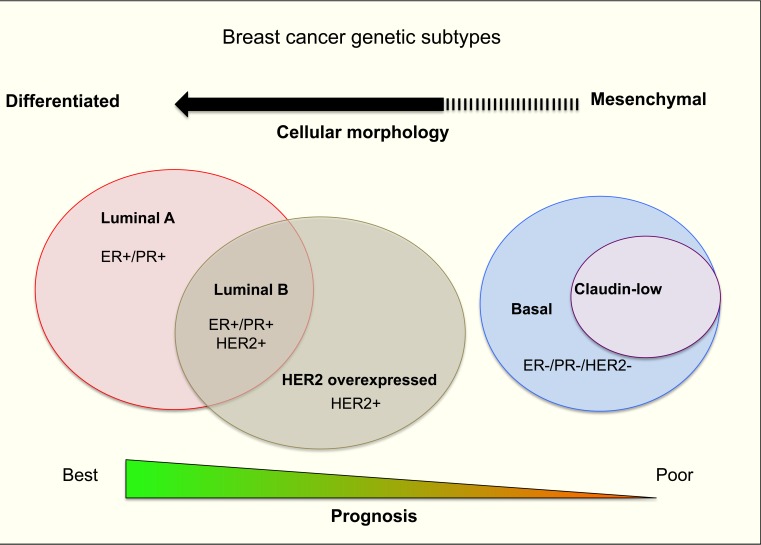
A summary of human breast cancer subtypes

Breast cancer (BrCa) accounted for 11.9% of total worldwide cancer deaths in 2012 [1] despite recent advances in treatment and surveillance. BrCa is a heterogenic disease that can be categorized into four main molecular distinct subtypes based on gene expression profiling: luminal A, luminal B, human epithelial growth factor receptor-2 (HER-2) overexpressing and basal/triple negative (TN) cancer subtype [2-5] (Figure 1). A slight majority of diagnosed BrCa cases are luminal A subtype [2, 4] distinguished by high oestrogen receptor (ER) and/or progesterone receptor (PR) expression, but with a low expression of the cell proliferation marker Ki-67 [2, 4].

## BRCA SUBTYPE MOLECULAR SIGNATURES AND ITS PROGNOSIS

Patients diagnosed with luminal A subtype have the best prognosis as this subtype is responsive to Tamoxifen hormone therapy [6]. The luminal B subtype also expresses ER and/or PR. However, as expression of the proliferation factor Ki-67 is higher than in the luminal A subtype, this subtype presents a higher risk of disease relapse [7]. HER-2 overexpressing subtype [2, 4], typically had a poor prognosis but the emergence of monoclonal antibody based therapies such as Trastuzumab, targeting the HER-2 receptor, has improved patient prognosis (44% reduction in risk of death) [8]. The basal/TN breast cancer subtype [2, 4] has the highest level of proliferation-related gene expression and frequencies of genetic mutations, such as *TP53* and *BRCA 1* [9]. Due to the lack of targeted treatments, the basal/TN subtype has the worst prognosis. Recently, a newly established breast cancer TN subtype, claudin-low, was described [2, 4] and was shown to lack epithelial cell-cell adhesion proteins such as E-cadherin and claudin 3, 4 and 7 [10]. Claudin-low tumours are also characterized by low luminal, high epithelial-to-mesenchymal transition features and by enhanced tumour initiating processes [11]. These properties render this subtype resistant to chemotherapy and hence these cells often dominate post-treatment tumour samples after neo-adjuvant chemotherapy or hormone therapy [12]. Patients diagnosed with this subtype also have a generally poor survival outcome [7]. Generally, each of the major subtypes comprises a roughly equal proportion of total breast cancer cases (11-23%).

**Figure 2:**
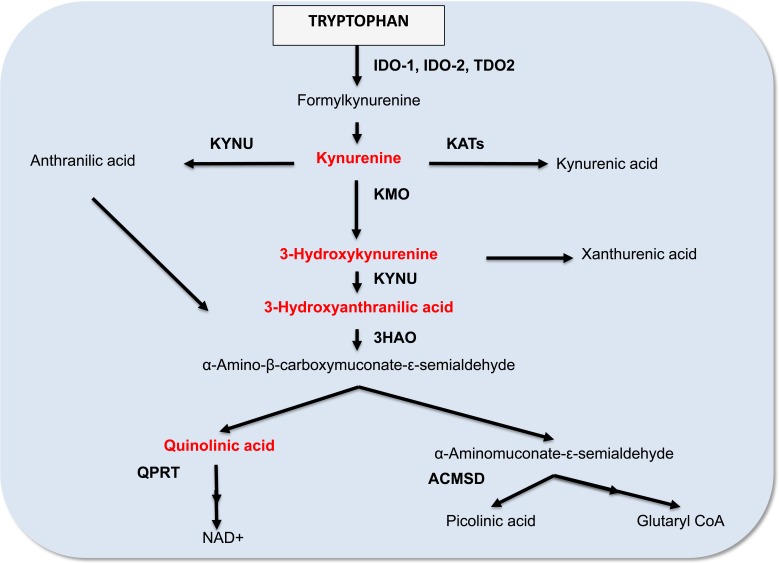
A simplified diagram of the kynurenine pathway

The molecular classification of breast tumour subtype has provided new opportunities to develop more appropriately targeted therapy. However, drug-based interventions will continue to be important for BrCa therapy. Another significant aspect to consider is the relationship between breast tumour development and immune tolerance. A particularly interesting recent development has been the discovery of the role of IDO1 in mediating tumour immune-evasion [13]. Specifically, alterations in tryptophan catabolism in both tumour and tumour-draining lymph nodes may provide a mechanistic avenue enabling tumour-cell persistence, a view that is supported by experimental evidence [14-16]. This review will focus on the contribution that alterations in tryptophan catabolism via the kynurenine pathway (KP; Figure 2) may play in BrCa progression. Understanding how BrCa cells exploit such immune evasion mechanisms may lead to identifying promising therapeutic targets for BrCa and metastasis based on modulation of tryptophan metabolism.

## TRYPTOPHAN METABOLISM: FOCUS ON THE KYNURENINE PATHWAY

Tryptophan is an essential amino acid obtained through the diet [17]. Under physiological conditions, the majority of tryptophan is catabolized through the KP to synthesize the vital energy cofactor, nicotinamide adenine dinucleotide (NAD^+^) [18]. Several downstream metabolites of the KP are biologically active in various physiological and pathological processes, including kynurenine (KYN), kynurenic acid, 3-hydroxykynurenine, anthranilic acid, 3-hydroxyanthranilic acid (3-HAA), picolinic acid and quinolinic acid (QUIN).

Three different heme-enzymes, indoleamine 2,3 dioxygenase 1 (IDO1) [19], indoleamine 2,3 dioxygenase 2 (IDO2) [20, 21] and tryptophan 2,3 dioxygenase (TDO2) [22], catalyse the first rate-limiting key step of the KP. Despite sharing the same substrate, the two IDO isoforms and TDO2 each have distinct inducers and patterns of tissue expression. IDO1 is highly induced by pro-inflammatory cytokines such as IFN-γ [23] whereas TDO-2 is induced by its substrate tryptophan and by glucocorticoids [24]. Induction of IDO2, however, is less well understood. IDO1 is commonly expressed in all major organs and immune T and B cells [25], whereas IDO2 is expressed by hepatocytes, in the bile duct, neuronal cells of the cerebral cortex and dendritic cells [26]. TDO-2 is primarily expressed in the liver [27], but is also expressed in placenta [28], maternal and embryonic tissues [29], and brain [30].

A key juncture of the KP leads to the catabolism of 2-amino-3-carboxymuconate semialdehyde (ACMS) to 2-aminomuconic acid 6-semialdehyde (AMAS) by 2-amino-3-carboxymuconate semialdehyde decarboxylase (ACMSD), then AMAS non-enzymatically converts to the neuroprotective metabolite picolinic acid (Figure 2) [31]. Alternatively, the KP can branch towards the non-enzymatic rearrangement of ACMS to form the metabolite QUIN, an essential precursor for *de novo* NAD^+^ synthesis. Under normal physiological conditions, the production of picolinic acid and QUIN is at equilibrium [32]. However, during chronic activation KP metabolism is diverted towards QUIN production, and hence NAD+ biosynthesis, which may promote cellular growth.

## KP-MEDIATED IMMUNE-MODULATION IN CANCER

**Figure 3:**
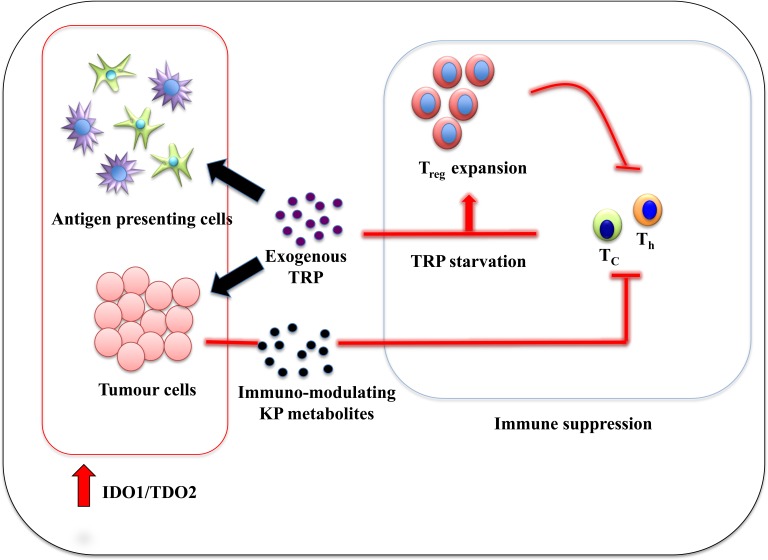
Immune tolerance mechanism by IDO1:TDO2 overexpression in cancer-associated inflammation environment

Since the demonstration that IDO1 has immuno-suppressive ability by mediating maternofetal tolerance [33], much attention has been dedicated to determining how IDO1 also modulates the immune response to tumours. In most forms of human cancers, including BrCa, high IDO1 expression is positively correlated to poor prognosis [34-41]. Studies utilizing murine cancer models confirmed that IDO1/TDO2 over-expressing tumours are more aggressive compared to tumours with basal IDO1/TDO2 levels [15, 16, 42]. In this paradigm, the generally pro-inflammatory cancer microenvironment leads to IDO1/TDO2 over-expression in stoma (epithelial and endothelial cells) and/or antigen-presenting cells (APCs) such as dendritic cells (DC) [43] resulting in depletion of tryptophan in the local milieu. Hence, local cytotoxic T-cells (T_c_) and T helper cells (T_h_) become tryptophan deficient leading to inhibition of proliferation as a result of general control nonderepressible 2 (GCN2) signalling pathway activation [44, 45]. Tryptophan starvation also predisposes activated T-cells to Fas-dependent apoptosis [44, 45]. Consequently, CD4^+^CD25^+^Foxp3^+^ regulatory T cells (T_regs_) mature to become the prominent T-cells population in the microenvironment [46-48]. These T-cell subset populations have a potent suppressive effect on both innate and adaptive immunity [49, 50]. Not only do T_regs_ impose immuno-tolerance on the microenvironment, but they also promote immuno-tolerance in draining lymph nodes, thereby potentiating the likelihood of distant metastasis, a phenomenon that has been observed in several cancer types and is a significant ongoing problem in BrCa (Figure 3).

*In vivo* studies using various animal models of human cancers treated with IDO inhibitors have provided relevant proof-of-concept that tryptophan metabolism is involved in tumour immune-escape. Two initial studies by Uyttenhove *et al.* [16] and Friberg *et al. [51]* demonstrated that the IDO inhibitor 1-methyltryptophan (1-MT), limited the growth of IDO1 overexpressing tumours engrafted in a syngeneic host. Further studies by Muller *et al. [42]* confirmed that 1-MT modestly slowed the growth of spontaneous mammary tumours in MMTV/neu mice. When combined with the cancer chemotherapeutic drug paclitaxel, MMTV/neu tumour size regressed by 30% with no side effects observed *[42]*. This suggested that 1-MT has only limited efficacy as a monotherapy, but is effective in combination together with chemotherapy. Additionally, immunodepletion of CD4+ or CD8+ T-cells before treatment abolished the efficacy of 1-MT treatment, suggesting that the anti-tumoral efficacy of 1-MT is dependent on a specific T-lymphocyte response.

While the majority of studies support the tumour-promoting activity of IDO1, it is important to note that other studies suggest that IDO1 may have anti-tumour activity *[52].* These studies demonstrated that IFN-γ induced IDO1 resulted in depletion of the essential metabolite tryptophan, presumably reducing NAD^+^ production within the tumour cell thereby limiting cell growth. Animal studies have shown that IFN-γ induced IDO1 restricted tumour growth [53, 54] and while clinical studies have shown that hepatocellular and renal cell carcinoma specimens evidenced a positive association between IDO1 expression and favourable outcomes [55, 56]. However, both clinical studies noted that IDO1 expression was restricted to tumour infiltrating cells but was not found in the tumour cells. This potentially highlights the different roles (pro- and anti-tumorigenic) of elevated IDO1 expression within tumour cells as well as its microenvironment.

## KP METABOLITES: INVOLVEMENT IN CANCER IMMUNOBIOLOGY

### KYN

KYN is the first catabolite produced from tryptophan by IDO1/2 and/or TDO2. KYN has been recently identified as an endogenous ligand for the aryl hydrocarbon receptor (AhR) [57], a key ligand-activated transcription factor involved in diverse cellular functions such as cellular differentiation and proliferation. Activation of the AhR by KYN promotes selective expansion of T_regs_ due to activation of forkhead box p3 (Foxp3) in naïve T-cells preventing maturation of T_h_17 cells [58]. Martin-Orozco and colleagues subsequently showed that depletion of the T_h_17 population favoured tumour growth in the lungs of mice injected intravenously with B16-F10 melanoma [59]. Adaptive transfer of antigen-challenged T_h_17 into tumour-bearing mice led to a lower rate of tumour establishment and growth whereas Th17 free mice could not prevent establishment of tumour allografts. The distinct anti-tumour effect of the T_h_17 cell subset reflects an ability to enhance DC infiltration and Tc cells immune response [59]. Accordingly, active expansion of T_regs_ cells through KYN-AhR activation creates an immune suppressive zone around IDO1 and/or TDO2 expressing tumours.

Enhanced tumour-cell survival and motility also results from KYN-AhR activation. Opitz *et al.,* found that TDO2 expressing (i.e. KYN producing) brain tumour xenografts in AhR-proficient mice had an enhanced tumour growth rate, increased levels of the inflammatory cytokines (IL-1β, IL-6 and IL-8) and a decreased number of infiltrating CD8+ cells around tumours with high AhR and TDO2 expression [57]. The persistence of elevated inflammatory cytokines in the microenvironment may lead to chronic IDO1 expression in APC cells such as macrophages and DC and/or tumour cells [60] thereby creating a pro-inflammatory feedback loop promoting tumour growth. Collectively, these studies highlight the importance of the AhR in IDO1 and/or TDO2 expressing tumours and emphasize the role that autocrine production of inflammatory cytokines plays in tumour survival and growth. Whether similar tumour-promoting interactions occur with KYN and AhR in BrCa remains unknown but are likely.

### 3-HAA

Among the KP metabolites, 3-HAA appears to have the highest capacity to modulate the immune functions of both monocytic cells and lymphocytes. Macrophages treated with 3-HAA, lose their ability to synthesize nitric oxide (NO), due to upstream inhibition of both NF-κB and inducible nitric oxide synthase (iNOS) activity [61]. Considering that NO production by macrophages is critical to their immune-mediator function, inhibition of NO synthesis may impair macrophage ability to eradicate tumour cells.

Induction of kinase 3-phosphoinositide-dependent protein kinase-1 [62], caspsase-8 and cytochrome *c* [63] by 3-HAA also induces apoptosis in both T_c_ and T_h_1 populations of effector T cell [64]. Additionally, 3-HAA also limits cytokine-stimulated Tc proliferation by reducing the number of T-cells entering the cell cycle [65]. The reduction of these two major effector T-cell populations may impair the immune response against KP expressing tumours. More recently, 3-HAA has also been shown to specifically enhance the differentiation of T_regs_. A 70% increase in the number of T_regs_ cells is observed after the treatment of naïve T cells with a KP metabolite cocktail containing 10 μM of 3-HAA [63, 64]. This result was further confirmed by both Favre *et al.* and Zaher *et al.* who also reported strong T_regs_ differentiation in the presence of 3-HAA [66, 67]. In summary, chronic production of 3-HAA by cancer cells may not only lead to progressive loss of competent immune cells but also expansion of immune suppressive cells, dampening immune surveillance and thereby encouraging tumour growth.

### Picolinic acid

Picolinic acid is one of the alternate end products of the KP, resulting from the enzymatic conversion of ACMS by the enzyme ACMSD (Figure 2). Picolinic acid is an endogenous metal chelator for elements such as iron [68]. Based on its iron chelation properties and the fact that other iron chelators such as desferrioxamine exhibit anti-tumour activity, picolinic acid has also been assessed for its anti-tumour activity [69]. Indeed, picolinic acid challenged tumour cells have decreased proliferation rates *in vitro* [70, 71] and *in vivo* [72], as compared to untreated control cells. Normal human cells, however, remain unaffected at the same dosage [73].

Picolinic acid is also associated with immune function. Picolinic acid interacts synergistically with IFN-γ to augment iNOS production by macrophages [74-76], increasing iNOS mRNA expression by 10 to 15 fold leading to a potent cytotoxic/cytostatic effect. This synergistic interaction was found to last for at least 20 hours. Picolinic acid challenged macrophages have been shown to inhibit tumour growth and increase survival in cancer animal models [76-78]. In addition to macrophage activation, picolinic acid also potently induces macrophage production of the chemokines macrophage inflammatory protein (MIP)-1α and -1β which also contributes to tumour eradication [79].

### QUIN

Under normal physiological conditions, QUIN is the precursor for the production of the essential co-factor NAD^+^. This reaction is catalysed by the QPRT (Figure 2). QUIN is an agonist of the N-methyl-d-aspartate (NMDA) receptor and in pathophysiological conditions excess QUIN can induce over-activation of the NMDA receptor leading to excitotoxicity in neurodegenerative disorders such as Alzheimer's disease [80]. Quin is also known to be involved in tumour neuropathogenesis and persistence [81-83]. When treated *in* vitro, Quin increased the proliferation rate of human glioblastoma U343MG cells [84], HT-116 and HT-29 colon cancer cells [85] and SK-N-SH neuroblastoma [71]. Interestingly, QUIN also increases astroglial production of glial cell line-derived neurotrophic factor [86], which may induce proliferation of cancer cells and increase resistance to chemotherapeutic agents [87]

We showed that QPRT is strongly elevated in higher grade gliomas leading to increased QUIN production, which was significantly associated with poor prognosis [88]. High QUIN levels promote tumour chemo-resistance due to enhanced production of NAD^+^, a vital substrate for Poly [ADP-ribose] polymerase-1 (PARP-1) activation [89], which facilitates repair of reactive oxygen species-induced DNA damage and enables cells to recover DNA replication after treatment [90]. In fact, QUIN treated malignant human glioma cells were less sensitive to temozolomide and H_2_O_2_ mediated apoptosis compared to untreated glioma cells [88]. Similarly to 3-HAA, QUIN also modulates the immune response through selective inhibition of T_h_ and T_c_ proliferation and natural killer cell activation [64]. QUIN expression can also increase T_regs_ population [67]. These results show that excessive QUIN in the tumour microenvironment may have a significantly detrimental effect on local immuno-surveillance. However, the precise role that QUIN may play in BrCa remains to be elucidated.

### Kynurenic acid

Kynurenic acid can antagonize QUIN excitotoxicity at NMDA receptors and hence functions as an endogenous neuroprotectant. Whereas 3-HAA and QUIN have immunosuppressive properties that are likely to promote tumour growth [81], kynurenic acid can inhibit adenocarcinoma and renal cell carcinoma cancer cell proliferation *in vitro* [91]. Inhibition of the mitogen activated protein kinase (MAPK) pathway proteins is one of the possible anti-tumour activities of kynurenic acid [91]. The MAPK pathway promotes several cellular processes including motility, proliferation and survival [92] and is frequently over-activated in human cancer [93, 94]. Kynurenic acid is also known to up-regulate the expression of p21 Waf1/Cip that induces cell-cycle arrest [95]. Hence the presence of kynurenic acid in the tumour microenvironment might help to limit tumour growth.

## REDOX MODULATION BY KP METABOLITES: POTENTIAL INVOLVEMENT IN CANCER GROWTH

Under certain intracellular or microenvironment conditions KP metabolites have been shown to generate reactive oxygen species (ROS) such as H_2_O_2_ and hydroxyl radical. Formation of ROS has been linked to cancer initiation and progression by inducing lipid peroxidation, DNA damage/mutation and cell proliferation [96]. Indeed, one of the most extensively studied LP products are the 4-hydroxy-2-nonenals (4-HNE) which modulate a number of signaling processes such as Akt pathway involved in cancer initiation and progression [97]

In the presence of transition metals such as copper (Cu^2+^), manganese (Mn^2+^) and iron (Fe^3+^), 3-hydroxykynurenine and 3HAA can be oxidized to generate ROS [98] which is a well-known cause of DNA damage [99]. More recently, it was suggested that 3-hydroxykynurenine mediated cell death independently of caspase-3 activation via p38 MAPK phosphorylation [100]. 3HAA-generated ROS was shown to enhance apoptosis in precursor immune cells such as thymocytes [101] and monocytes [102]. Furthermore, 3HAA can auto-oxidize and generate cinnabarinic acid, which induces apoptosis in thymocytes by an order of magnitude greater than 3HAA [101]. Redox-mediated depletion of these precursor immune cells by 3HAA could lead to tumor persistence due to reduced numbers of mature T-cells. Despite much research supporting a pro-oxidant effect, 3-hydroxylkynurenine and 3HAA also have documented antioxidant properties [103-105]. In fact, 3-hydroxykynurenine and 3HAA has been shown to protect the brain and gliomas against oxidative stress by inhibiting spontaneous lipid peroxidation [106, 107].

QUIN is another KP metabolite that enhances ROS formation in the tumour microenvironment by several mechanisms including formation of redox-active complexes with Fe^2+^ leading to lipid peroxidation [108]. However, QUIN was also observed to act as an anti-oxidant in electrochemical studies. Low concentrations of QUIN reduced the rate of ROS production by affecting the Fe^2+/^Fe^3+^ ratios, only at concentrations above pathophysiological levels were pro—oxidant effects observed [109].

These somewhat conflicting observations suggest that the specific environmental milieu may determine the net ROS balance of these metabolites, which is not yet precisely defined in cancer.

## EVIDENCE FROM CLINICAL STUDIES

Up-regulation of KP metabolism in BrCa patients was first reported by Rose *et al* (1967) who found increased kynureninase (KYNU), kynurenine-3-monooxygenase (KMO), and kynurenine aminotransferase-II enzyme activity (Figure 2) in the urine of untreated BrCa patients compared to controls [110]. While one-third of the patient cohort exhibited higher activity of these three enzymes, the remaining patients had similar enzymatic profiles to the control group. A potential explanation may be that the KP is differently modulated in the different BrCa subtypes. A later study by Sakurai *et al.* also detected higher IDO1 activity in the serum of BrCa patients and higher IDO1 mRNA expression in BrCa compared to normal tissue [111]. The elevated serum levels and IDO1 expression in tumour correlates to clinical stage, in agreement with another study by Rose *et al.* showing elevated KP enzyme activity in approximately one third of early cancer patients and in half of advanced BrCa patients respectively [112]. However, increased KP activity in advanced-stage patients was only evident in those with soft tissue metastases. Patients with skeletal metastases displayed normal KP activity, suggesting that KP over-activation may drive proximal tissue invasion but not distal metastasis. There is also evidence of immune system suppression in IDO1-positive advanced BrCa patients as IDO1 expressing breast tumours had higher numbers of infiltrating T_regs_ in tumour and lymph node metastases [113-115].

A later prospective cohort study showed that KP metabolism returned to near normal levels in BrCa patients post-mastectomy, with- or without-oophorectomy, further demonstrating the link between tumour presence and KP modulation [116]. More recently, post-surgical re-normalization of KP metabolism in BrCa patients was confirmed together with reduced IDO1 activity expression post-chemotherapy [117].

Despite much evidence that IDO1 expression is associated with poor prognosis in BrCa patients, a study of medullary BrCa patients showed that high IDO1 expression was associated with favourable outcomes [118]. This study agrees with others [55, 56] where IDO1 expression was observed in the surrounding dendritic-like monocytes rather than the tumour cells. This again raises the possibility that IDO1 locality determines whether it supports or prevents tumour growth.

### Evidence from microarray databank

#### Data mining from ex vivo clinical studies

An early collective and global BrCa study in cBioportal using the Agilent microarray series showed that a total 86.3% of all BrCa cases had altered mRNA expression of enzymes in the KP (454 out of 526 BrCa patients, z-score of −1< and *>*1, Table 1A) [119, 120]. Data from the Cancer Genome Atlas (TCGA) confirmed the cBioprotal results and showed a slightly higher percentage of total 91.4% of patients with altered KP enzyme expression (169 of 185 patients, z-score of −1< and >1; Table 1A). However, as both databases did not differentiate BrCa subtype, it is not possible to draw firm conclusions regarding whether specific KP enzymes were up-or down-regulated. Furthermore, the heterogeneous database presentation may dilute subtype specific KP dysregulation evidence.

**Table 1A:** KP enzymes mRNA expression by microarray database

	cBioPortal (*n* = 526)		TCGA (*n* = 185)	
	%. of KP mRNA changes	% of elevated mRNA	%. of KP mRNA changes	% of elevated mRNA
**IDO-1**	N.A.	N.A.	32	53
**TDO2**	33	53	34	51
**KMO**	35	61	36	52
**3HAO**	22	49	21	53
**KYNU**	26	55	23	60
**ACMSD**	22	49	24	80
**QPRT**	36	54	37	49

However, microarray data from patients with invasive breast carcinoma (cBioPortal; PAM50 series) [119, 120] (Table 1B, z score <-1 and >1) overcame this limitation. This data showed that the mRNA expression of specific KP enzymes differed substantially by BrCa subtype. Significantly, the claudin low subtype, exhibited elevated TDO2 and KYNU mRNA expression in more than half of the samples; while a quarter of the samples exhibited elevated KMO and 3-hydroxyanthranilate 3,4-dioxygenase (3HAO). In the similarly aggressive basal BrCa subtype, both TDO2 and KYNU were also elevated. Elevated expression of TDO2 is indicative of active breakdown of tryptophan leading to increased production of the potent immuno-suppressive metabolite 3-HAA. Intriguingly, ACMSD remained unchanged or down-regulated in these subtypes suggesting a KP pathway shift towards quinolinic acid and energy production (thereby facilitating tumour progression) and away from production of the tumour suppressive metabolite picolinic acid. In the HER2-overexpressing BrCa subtype, TDO2, KMO, KYNU and QPRT were elevated similarly to both the claudin-low and basal subtypes in 34% to 59% of total specimens. Considering that these subtypes are associated with higher rates of lymph node metastasis, elevation of the early KP enzymes TDO2, KMO and KYNU may promote tumour aggressiveness and metastasis. Interestingly, a portion of HER2 over-expressing specimens had elevated ACMSD, suggesting increased production of anti-tumour picolinic acid metabolite. This is a potentially contradictory observation in a subtype associated with poor prognosis, given the anti-tumour activity of picolinic acid (as described above) anti-tumorigenic property. However, a potential explanation of this anomaly may be that enhanced picolinic acid by tumour cells could be a potential strategy to chelate additional iron for growth, although there is no data to support this hypothesis.

**Table 1B:** KP enzymes mRNA expression by human breast cancer subtypes from PAM50 series/cBioPortal

	Claudin low (*n* = 8)		Basal (*n* = 81)		HER2 (*n* = 58)		Luminal A (*n* = 235)		Luminal B (*n* = 133)	
mRNA changes	Total changes (%)	Elevated (%)	Total changes (%)	Elevated (%)	Total changes (%)	Elevated (%)	Total changes (%)	Elevated (%)	Total changes (%)	Elevated (%)
**TDO2**	63	100	34	79	41	92	29	25	34	56
**KMO**	25	100	12	60	59	100	36	57	35	49
**3HAO**	25	100	22	67	19	82	21	55	26	17
**KYNU**	63	100	27	86	34	95	23	34	25	39
**ACMSD**	38	0	20	0	19	64	28	89	25	76
**QPRT**	13	100	31	56	55	94	31	31	35	66

In contrast, both the low-metastatic risk luminal A and B subtypes showed no changes in KP apart from elevated ACMSD mRNA, which as discussed, could lead to higher concentrations of picolinic acid and a more tumour suppressive KP profile.

Clinical data from the Genes-to-Systems Breast Cancer database [121] was then used to examine the relationship between breast tumour grade and KP mRNA expression profile. This analysis showed that TDO2 expression was higher in well, and moderately differentiated grade 1 and 2 breast tumours, compared to poorly differentiated grade 3 tumours. Increased KP enzymes mRNA expression in invasive breast carcinoma was also observed in the European Molecular Biology laboratory Gene Expression Atlas (EMBL; Table 1C) [122-125]. All invasive carcinoma samples examined in these studies had elevated IDO1, TDO2 and KMO. Interestingly, normal breast epithelial cells in the immediate tumour proximity also showed elevated TDO2 and KMO expression, implying that IDO1/TDO2 expressing tumours have the capability to influence KP activity in surrounding normal tissue. This not only highlights the potential interplay between these three KP enzymes in facilitating tumour invasion, but also suggests that targeting a single KP enzyme may not be optimal for complementary cancer immunotherapy. This agrees with our study demonstrating that the entire pathway is active in human brain tumours that suppresses the anti-tumour immune response and support tumour growth [57]. Collectively, these data suggest that the elevated KP activity contributes to tumour aggressiveness.

**Table 1C:** KP enzymes mRNA expression in human breast cancer specimens from EMBL-gene Atlas

Disease group	n value	KP enzymes expression			Array number	Ref.
		IDO-1	TDO2	KMO		
**Normal breast**	143				E-GEOD-10780	96
**Invasive ductal carcinoma**	42					
**Reduction mammoplastices**	10				E-TABM-276	97
**Invasive ductual carcinoma**	23					
**0 cm away from tumour**	28					
**1 to 4 cm away from tumour**						
**Normal breast**	15				E-GEOD-8977	98
**Invasive ductal carcinoma**	7					
**Ductal carcinoma in situ**	53				E-GEOD-41194	99
**Invasive breast cancer**	51					

*Black box: No change in mRNA expression; Green box: up regulation in mRNA expression

## CONCLUSIONS

Since the discovery of the essential role played by IDO1 in mediating maternal foetal tolerance, a great deal of interest has focused on the roles that IDO1 and other KP enzymes may play in cancer immunology. Over the past decade strong evidence has accumulated that confirms that IDO1/TDO2 overexpression in tumour results in tryptophan depletion in the microenvironment, in turn, suppressing the T-cell mediated immune response. Other observations also implicate KP activation in promoting immune evasion. Higher 3-HAA concentrations, resulting from increased KYNU activity, causes reduced iNOS expression in macrophages, impairing their anti-tumour activity. Increased Tregs population caused by KYN-AhR activation further promotes immune suppression in the tumour vicinity and increases in QUIN, the NAD^+^ precursor, enhances cell proliferation. Considering these observations, there has been considerable interest in the potential of IDO1 inhibitors to reverse immune evasion with the majority of studies providing positive results.

Clinical data confirms the role of KP activation in BrCa and provides support for the use of KP enzyme inhibitor/s in addition to standard chemotherapy regimens. Significantly, KP activation seems to be associated with the more aggressive forms of BrCa that readily metastasize. Hence, it is possible to speculate that BrCa patient KP profiling may provide a valuable biomarker potentially capable of discriminating between non-invasive and invasive BrCa. Such methodology may have significant diagnostic, prognostic and/or therapeutic value.

## Abbreviations

1-MT: 1-methyltryptophan
3-HAA: 3-hydroxyanthranilic acid
3HAO: 3-hydroxyanthranilate 3,4-dioxygenase
ACMS: 2-amino-3-carboxymuconate semialdehyde
ACMSD: 2-amino-3-carboxymuconate semialdehyde decarboxylase
AhR: Aryl hydrocarbon receptor
AMAS: 2-aminomuconic acid 6-semialdehyde
APCs: Antigen presenting cells
BrCa: Breast cancer
DC: Dendritic cells
ER: Estrogen receptor
Foxp3: forkhead box p3
IDO1: Indoleamine 2,3 dioxygenase 1
IDO2: Indoleamine 2,3 dioxygenase 2
iNOS: inducible nitric oxide synthase
KMO: Kynurenine-3-monooxygenase
KP: Kynurenine pathway
KYN: Kynurenine
KYNU: Kynureninase
MAPK: mitogen activated protein kinase
NAD+: Nicotinamide adenine dinucleotide
NMDA: N-methyl-d-aspartate
NO: Nitric oxide
QUIN: quinolinic acid
PR: Progesterone receptor
ROS: Reactive oxygen species
Tc: Cytotoxic T-cells
TDO2: Tryptophan 2,3 dioxygenase 2
Th: T helper cells
TN: Basal/triple negative
Tregs: CD4+CD25+Foxp3+ regulatory T cells
